# Infantile pustular psoriasis: Case report of successful treatment with acitretin in a 4-week-old infant

**DOI:** 10.1016/j.jdcr.2021.03.038

**Published:** 2021-04-01

**Authors:** Shantanu Srivatsa, J. Scott Boswell, Thaddeus W. Mully

**Affiliations:** a Duke Global Health Institute, Duke University, Durham, North Carolina; b Boswell Dermatology, Fresno, California; c UCSF Dermatopathology and Oral Pathology Service, San Francisco, California

**Keywords:** acitretin, infantile pustular psoriasis, retinoid, IPP, infantile pustular psoriasis

## Introduction

Generalized pustular psoriasis is a severe variant of psoriasis characterized by scattered pustules on top of underlying erythematous skin, distributed across the trunk and extremities.^1^ Infantile pustular psoriasis (IPP) is extremely rare and thought to account for approximately 0.6% of pustular psoriasis cases.^1^ However, early diagnosis and management of infantile cases is critical to avoid life-threatening complications, such as bacterial super-infection and sepsis. Infantile pustular psoriasis presents a therapeutic challenge due its rarity, risk for adverse outcomes, and lack of evidence as to best practices for treatment.^2^ The scarcity of cases has resulted in a large portion of current clinical evidence being sourced from case reports and series, rather than large observational studies or randomized trials.^3^ Systemic retinoid treatment, particularly acitretin, has been proposed and shown efficacious as a first-line treatment for pustular psoriasis.^4^ Here, we report a case of a 4-week-old infant with IPP who was successfully treated using acitretin. To our knowledge, this is the youngest patient with IPP in the extant literature to respond successfully to systemic retinoid treatment.

## Case report

**Fig 1:**
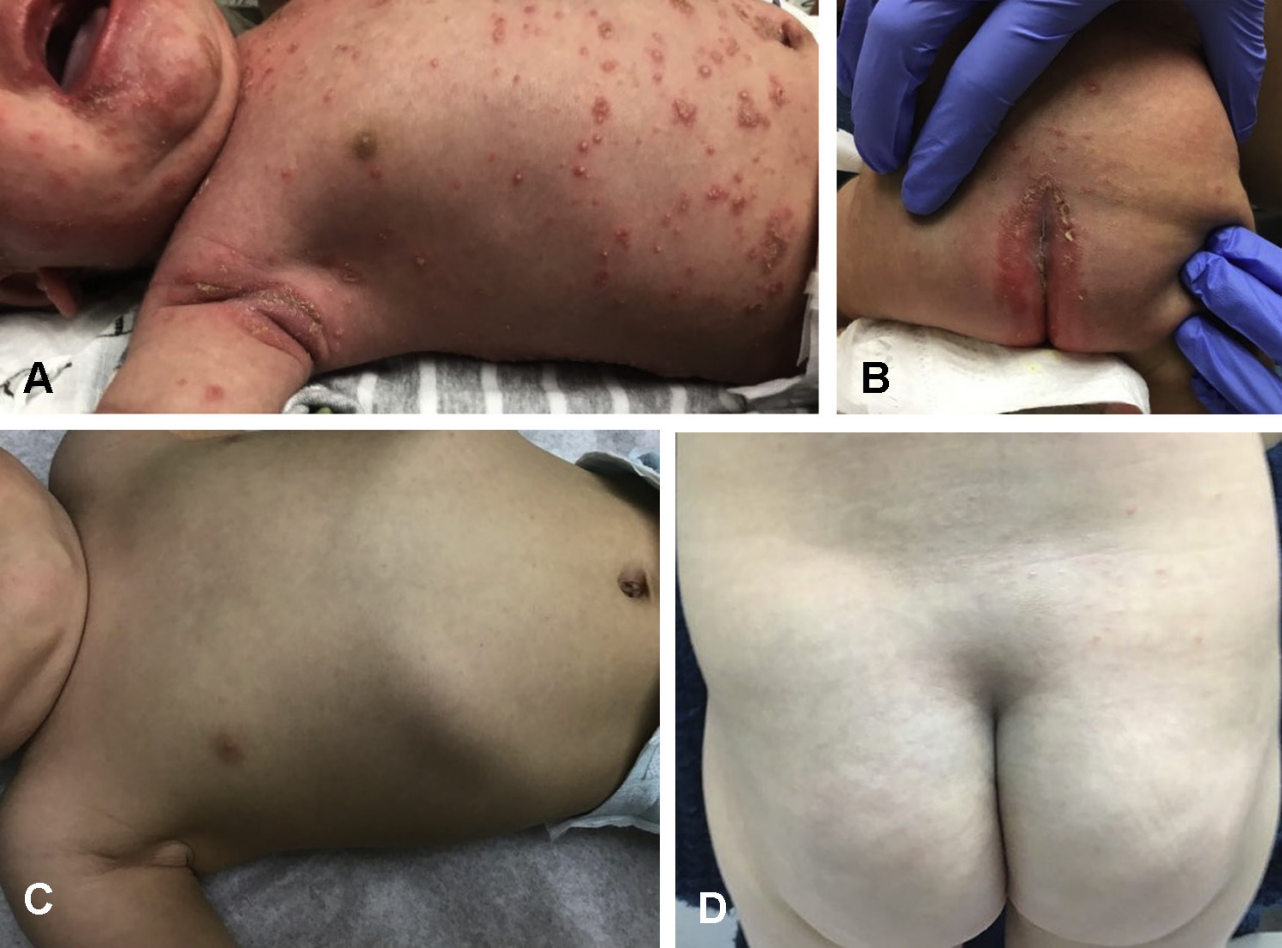
Upon presentation, a 4-week-old infant presented with generalized pustules, which evolved into psoriasiform, exfoliative plaques, involving the (**A**) arms, face, axillae, and trunk and (**B**) flexural areas (gluteal cleft). Considerable clearing was evident in the same areas (**C**) and (**D**) after 6 months of acitretin treatment.

**Fig 2:**
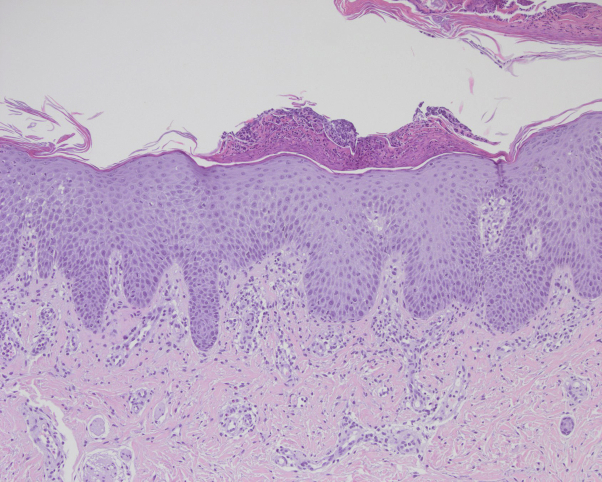
Histology revealed a sparse perivascular lymphocytic infiltrate, psoriasiform epidermal hyperplasia with attenuation of the granular layer, and parakeratosis, which contained collections of neutrophils. The blood vessels at the tips of the dermal papillae were dilated. An increased number of epidermal mitotic figures were also noted.

A 4-week-old infant boy presented to the clinic with a concerning rash developing over 5 days. Clinical examination revealed generalized erythema and desquamation around the axillae, face, genitalia, lip, and trunk. Dozens of pustules were developing on the arms, face, legs, flexural areas, and trunk (Fig 1, *A* and *B*). No fever, hepatomegaly, or splenomegaly was noted on exam. The patient received the typical injections shortly after birth, including the first dose of the hepatitis B vaccine and a vitamin K injection. There was no maternal or family history of psoriasis or gestational pemphigoid, and the pregnancy and the patient's delivery were both without complications. Due to the presentation of exfoliative dermatitis and diffuse erythema, as well as the patient's young age, the patient was admitted for sepsis workup and intravenous antibiotics for suspected staphylococcal scalded skin syndrome. The patient was unresponsive to oral and intravenous antibiotics, and fungal, viral, and bacterial cultures taken from the pustules were negative. Because the rapid evolution of the pustules into psoriasiform plaques over two days strongly suggested psoriasis, direct immunofluorescence, genetic, and immunodeficiency tests were not performed. Subsequent biopsy revealed psoriasiform epidermal hyperplasia, dilated blood vessels within dermal papillae, and parakeratosis with aggregated neutrophils (Fig 2), all of which findings were consistent with pustular psoriasis, and a diagnosis of IPP was established. The patient received topical fluocinolone acetonide 0.01% oil and triamcinolone acetonide 0.025% ointment and was discharged, but returned within a week with increasing pustules. Due to the worsening condition despite topical therapy and risk for electrolyte imbalance and readmission to the hospital, oral acitretin treatment was initiated, following normal baseline laboratory findings (complete blood count with differential, hepatic, and lipid panel). Given that acitretin has been shown successful in the literature at a dosing of 0.5-1.0 mg/kg/day, the patient was started at 2.5 mg acitretin (0.56 mg/kg/day).^5^ The use of acitretin in infants presents unique challenges due to commercial availability of 10 mg and 25 mg pills, and the much smaller dosing amounts required. In concordance with the literature and pharmaceutical recommendations, 10 mg capsules were prescribed with instructions to place them in the freezer, cut ¼ pill daily (discarding the remaining ¾ due to acitretin's photodegradation), and dissolve the ¼ pill in 2 ounces of expressed breast milk (EBM) or formula daily. With this daily regimen of 2.5 mg, the patient's pustular and exfoliative psoriasis cleared over a 6-month period, with regular monitoring of his complete blood count, hepatic, and lipid panel (Fig 1, *C* and *D*). Thereafter, acitretin was tapered to 2.5 mg once every 3 days for another 4 months. At 10 months, treatment was discontinued due to remission for months other than a mild flare-up, which responded to topical steroid therapy.

## Discussion

Infantile pustular psoriasis is extremely rare among presentations of juvenile psoriasis, with one case review finding pustular psoriasis to account for only 9.8% of total psoriasis cases in patients <2 years of age.^6^ Early diagnosis and management are crucial for infantile cases, as uncontrolled IPP may lead to adverse complications such as infection and sepsis. Systemic retinoid treatment can be used as first-line therapy for infants with pustular psoriasis, with reports of patients as young as 6 weeks old successfully treated.^7^ Although there are concerns over skeletal toxicity with long-term retinoid use in young patients, studies have shown that IPP patients tend to respond remarkably fast and tolerate retinoid use well, with one study following a child for 3 years after completed treatment, with no adverse side effects observed.^5^

To our knowledge, the successful treatment and safe use of acitretin in a 4-week-old infant described herein is the youngest reported in the literature. Analysis of case studies shows that successful dosing ranges from 0.5-1.0 mg/kg/day, with tapering after 3-4 months of successful treatment to ⅓-½ of the initial dose.^5^^,^^8^ Remission for the patient was achieved at 10 months using acitretin at 0.56 mg/kg/day. Other infantile cases ranging in severity have had recovery as fast as 6 weeks with higher dosing (1 mg/kg/day) up to a year for more recalcitrant cases.^1^^,^^5^

In addition, we describe a method of drug delivery more appropriate for infantile patients. Dissolving freshly cut, frozen acitretin capsules in either EBM or formula is an advised method for neonatal or infantile patients, as it allows for safe consumption of the medication in conjunction with normal daily feeding. When acitretin capsules are frozen and cut, parents should be advised to discard the remainder rather than save and use the remaining fraction over the following days. Acitretin as a compound has reduced stability in acidic or photosensitive environments, which may lead to reduced efficacy. Since there are no standardized guidelines around acitretin use in infants, we suggest that this method may be appropriate for other providers seeking to use acitretin for infantile cases.

Acitretin should be considered as a first-line medication for cases of IPP that fail topical therapy, with several studies documenting its fast action, safety, and efficacy.^1^^,^^5^^,^^7^^,^^8^ Here, we show its safe and successful use in a 4-week-old infant, in whom it induced complete remission after 10 months of therapy. As newer biologic medications focus on inhibiting specific cytokines (eg, interleukin 17 and interleukin 23) for psoriasis, our case emphasizes the still time-proven utility of acitretin in pustular psoriasis variants, including IPP.

## Conflicts of interest

None disclosed.
